# Existence of Initial Dip for BCI: An Illusion or Reality

**DOI:** 10.3389/fnbot.2018.00069

**Published:** 2018-10-26

**Authors:** Keum-Shik Hong, Amad Zafar

**Affiliations:** ^1^School of Mechanical Engineering, Pusan National University, Busan, South Korea; ^2^Department of Cogno-Mechatronics Engineering, Pusan National University, Busan, South Korea

**Keywords:** initial dip, neuronal firing, vector phase analysis, brain–computer interface (BCI), functional near-infrared spectroscopy (fNIRS), functional magnetic resonance imaging (fMRI), intrinsic signal optical imaging (ISOI)

## Abstract

A tight coupling between the neuronal activity and the cerebral blood flow (CBF) is the motivation of many hemodynamic response (HR)-based neuroimaging modalities. The increase in neuronal activity causes the increase in CBF that is indirectly measured by HR modalities. Upon functional stimulation, the HR is mainly categorized in three durations: (i) initial dip, (ii) conventional HR (i.e., positive increase in HR caused by an increase in the CBF), and (iii) undershoot. The initial dip is a change in oxygenation prior to any subsequent increase in CBF and spatially more specific to the site of neuronal activity. Despite additional evidence from various HR modalities on the presence of initial dip in human and animal species (i.e., cat, rat, and monkey); the existence/occurrence of an initial dip in HR is still under debate. This article reviews the existence and elusive nature of the initial dip duration of HR in intrinsic signal optical imaging (ISOI), functional magnetic resonance imaging (fMRI), and functional near-infrared spectroscopy (fNIRS). The advent of initial dip and its elusiveness factors in ISOI and fMRI studies are briefly discussed. Furthermore, the detection of initial dip and its role in brain-computer interface using fNIRS is examined in detail. The best possible application for the initial dip utilization and its future implications using fNIRS are provided.

## Introduction

Over the last few decades, researchers in the neuroscience field have made significant advances in decoding thoughts based on brain activities. A complete understanding of the underlying neuronal mechanisms and signaling pathways is required to decode the brain. Therefore, interest in understanding the neurovascular coupling (NVC) has rapidly grown over the last years. NVC can be defined as a process that links the change in regional neuronal activity to the local cerebral blood flow (CBF) (Raichle, 1998). A tight coupling exists between the changes in the neuronal activity and the changes in CBF caused by functional stimulation (Villringer and Dirnagl, 1995), which is the basis for currently available functional neuroimaging techniques, including position emission tomography (PET) (Terpogossian et al., 1975; Fox and Raichle, 1984; Ollinger and Fessler, 1997), intrinsic signal optical imaging (ISOI) (Grinvald et al., 1988), functional magnetic resonance imaging (fMRI) (Ogawa et al., 1990; Bandettini et al., 1992), and functional near-infrared spectroscopy (fNIRS) (Hoshi and Tamura, 1993; Villringer et al., 1993; Kim et al., 2017).

Position emission tomography requires radioactive isotopes to monitor the hemodynamic response (HR) and metabolic changes associated with the neuronal activity, which limits its use in terms of continuous and repeated measurements (Hillman, 2007; Irani et al., 2007). ISOI is a minimally invasive technique used to measure the changes in HR [i.e., oxy-hemoglobin (HbO), deoxy-hemoglobin (HbR), and total hemoglobin (HbT)] by recording the images of the cortex illuminated by different wavelengths with a charged-couple device camera (Tso et al., 1990). fMRI is a non-invasive technique for measuring blood oxygenation level-dependent (BOLD) changes that rely on the HbR changes acting as an endogenous paramagnetic contrast agent (Kwong et al., 1992). fNIRS is a relatively new and low-cost non-invasive technique that uses near-infrared light of two wavelengths to simultaneously determine the changes of oxy-hemoglobin (ΔHbO) and deoxy-hemoglobin (ΔHbR) (Kato et al., 1993). In comparison to other existing techniques like electroencephalography (EEG), electrocorticography, magneto-encephalography, and single unit recording, the aforementioned HR-based functional imaging techniques have an advantage of mapping a large population of neurons at a high spatial resolution (Vanzetta and Grinvald, 2008). However, all these techniques are indirect measurements of neuronal activity; therefore, understanding their relation to the neuronal activity is crucial for the correct interpretation of the functional brain-imaging data.

**FIGURE 1:**
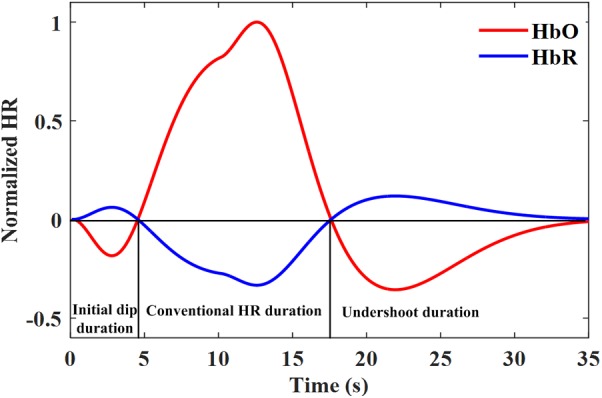
A typical hemodynamic response including the initial dip modeled by three gamma functions assuming a 10 s task.

HR-based modalities use the increase of blood flow (HbO/ΔHbO or/and HbR/ΔHbR) as an indirect marker for neuronal activity (Ances, 2004). However, the spatial and temporal characteristics of the HR as an alternative to the neural activity are still being debated (Logothetis et al., 2001; Ugurbil et al., 2003; Hillman, 2014). The HR caused by functional stimulation is mainly categorized into three response components (Frostig et al., 1990; Ernst and Hennig, 1994): (i) An initial increase/decrease in HbR/HbO caused by the extraction of oxygen by nearby active neurons known as the initial dip; (ii) the positive/negative response of HbO/HbR, which is also known as the conventional HR, caused by a large increase in CBF; and (iii) the undershoot, which is a decrease in response below the baseline. Figure 1 shows the schematic of a typical normalized HR depicting the initial dip, main HR period, and undershoot period. This HR was modeled by three gamma functions assuming a 10 s task (Shan et al., 2014). The initial dip response is believed to be a faster and better spatial localizer on the neuronal activity than the delayed HR because it is originated from the initial increase (or decrease) in HbR (or HbO) due to the increase in metabolism followed by the increase in CBF (Duong et al., 2000; Kim et al., 2000a).

These neuroimaging modalities can also be used to provide patients a means of communication with the real world through a brain–computer interface (BCI). The main role of the BCI is to translate brain signals and generate reliable commands with high accuracy to control external devices like a robotic arm/leg or a wheelchair in a real environment for patients (Mcfarland and Wolpaw, 2010, 2011; Nicolas-Alonso and Gomez-Gil, 2012; Ortiz-Rosario and Adeli, 2013; Muller-Putz et al., 2015; Hong et al., 2018a). Among all HR-based modalities, fMRI and fNIRS can be used for non-invasive BCI applications (Naito et al., 2007; Matthews et al., 2008; Sitaram et al., 2009; Sokunbi et al., 2014). fMRI has a constraint being bulky; therefore, it cannot be used as a portable device. However, it can be used for training patients to learn self-regulation of specific brain areas (Sitaram et al., 2008). In comparison to fMRI, fNIRS has a great potential to be used for BCI applications because of its high temporal resolution, low cost, and portability (Naseer and Hong, 2015; Vansteensel et al., 2017). The main limitation of HR for BCI is its slow nature and inherent onset delay (Jasdzewski et al., 2003; Cui et al., 2010; Ahn and Jun, 2017). A possible solution for coping up with this delay is the utilization of initial dip detection for fast fNIRS-based BCI applications (Hong and Naseer, 2016).

This paper presents a review focusing on initial dip detection during the HR. First, the existence of initial dip and its elusive nature will be briefly discussed using ISOI and fMRI. We will then discuss in detail the existence of the initial dip using fNIRS and its role in BCI. Some possible contributing factors in the initial dip elusiveness, future implications, and extensions will also be presented.

## What Is Initial Dip?

In ISOI, the onset of a neuronal activity-dependent oxygen delivery was first found to occur before the increase in the cerebral blood volume (CBV) and the CBF (Frostig et al., 1990). Upon activity, the HbR starts to increase, followed by a later and more pronounced decrease. This initial increase in the HbR is caused by the early extraction of oxygen by locally metabolized neurons from the capillary network before the vasculature provides more oxygenated blood to the active spot resulting in the CBV and the CBF to increase (Malonek and Grinvald, 1996). This early increase in the HbR is highly spatially coincident with the area of neuronal activity as compared to the delayed and prolonged decrease/increase in the HbR/CBV. In fMRI, the initial increase in the HbR at the beginning of the neuronal activity subsequently leads to an early decrease in the BOLD signal (Menon et al., 1995). This early decrease in the BOLD signal was first termed as “initial dip” in the fMRI research community. Similarly, in fNIRS, an initial decrease/increase in the ΔHbO/ΔHbR upon activity represents the initial dip (Kato et al., 1999; Kato, 2004).

## Existence of Initial Dip in Different HR Modalities

**FIGURE 2:**
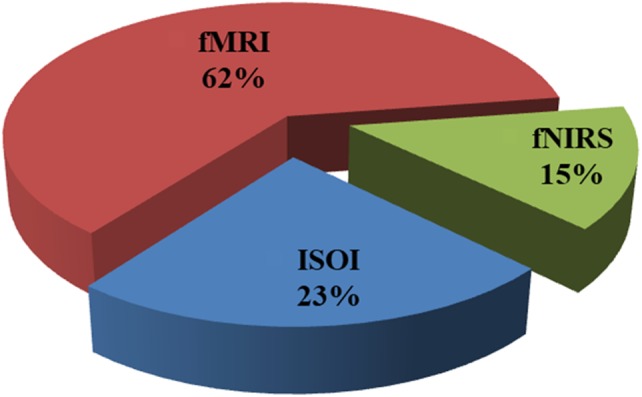
Percentage breakdown of the articles on initial dip (1990–2018): total number of articles was 103 (ISOI: 24, fMRI: 64, fNIRS: 15) from Web of Science (www.isiknowledge.com).

Figure 2 shows the percentage of articles published on the initial dips appearing ISOI, fMRI, and fNIRS during 1990 to 2018 from the Web of Science, http://isiknowledge.com. The initial dip existence and its elusive nature in each modality are discussed herein separately in the subsequent sections.

### Initial Dip in ISOI

After the inaugural work on the existence of an initial dip (Grinvald et al., 1991), Malonek and Grinvald (1996) did further in-depth analysis to study the activity-dependent changes in the HbO and the HbR in the visual cortex (brain area 18) of an anesthetized cat. They found that the HbR transiently increased and highly spatially confined to the neuronal active site. In comparison to the HbR, the delayed HbO and CBV increase was found to be less localized to the neuronal activity site. The authors interpreted that this initial increase in HbR was caused by an increased oxygen demand from the activated neurons (i.e., an increase in the neuronal activity could lead to a local increase in the oxidative metabolism). The initial dip can be expected to highly localize to the neuronal activity because of the local increase in metabolism. To compensate for this increased oxygen demand, more oxygenated blood comes into the active site, resulting in an increase in the CBV and the CBF (i.e., like “watering the garden for the sake of one thirsty flower”).

The controversy was started by raising doubt on the type of analysis used by Malonek and Grinvald (1996). They used the classical Beer–Lambert model to estimate the stimulus-evoked changes in the HbO and the HbR without considering the wavelength dependency on the optical pathlength. After which, several groups investigated the effect of optical pathlength on the initial dip by employing more rigorous spectroscopic analysis models in rats (Mayhew et al., 1999, 2000; Nemoto et al., 1999; Kohl et al., 2000; Jones et al., 2001; Lindauer et al., 2001; Sheth et al., 2004, 2005; Suh et al., 2006b). Unfortunately, these groups came up with conflicting conclusions, resulting in the elusive nature of the initial dip. Initial dip was detected in the spectroscopic data of one group (Mayhew et al., 1999, 2000; Jones et al., 2001; Sheth et al., 2004), but was not observed in the data of other groups using the same methodology (Kohl et al., 2000; Lindauer et al., 2001).

Similarly, the differences also existed in the optical studies related to the concurrent decrease in the HbO with the increase in the HbR that affected the interrelationship between the changes in oxygen metabolism, CBV, and capillary recruitment (Buxton, 2001). The decrease in the HbO was found in some studies (Devor et al., 2003, 2005), but not in others (Dunn et al., 2005; Devor et al., 2007). Therefore, the other techniques [e.g., oxygen phosphorus quenching (Vanzetta and Grinvald, 1999) and direct measurements of the changes in the partial pressure of tissue oxygen (pO_2_) using an O_2_ microelectrode (Ances et al., 2001; Thompson et al., 2003)] were further used to find hypo-oxygenation (i.e., decrease in the HbO). The oxygen phosphorous quenching technique measures the functional stimulation driven changes in oxygen concentration within the microvasculature. Using the oxygen phosphorous quenching technique, Vanzetta and Grinvald (1999) detected the initial hypo-oxygenation in a cat. Lindauer et al. (2001) used the same recording method, but did not find hypo-oxygenation in a rat. However, in the case of direct measurements of pO_2_, a transient decrease in pO_2_ was repeatedly observed before the CBF increase both in a rat and a cat (Ances et al., 2001; Thompson et al., 2003). This deoxygenation (i.e., a transient decrease in pO_2_) was better localized in the spiking activity than the later phase of the response (Thompson et al., 2004, 2005); thereby further conforming to the findings on the initial dip.

Using ISOI, the initial dip was also found in a monkey (Shtoyerman et al., 2000) and in humans (Suh et al., 2006a). An early decrease/increase in the HbO/HbR was observed in humans, which was spatially focused in the stimulated gyrus (Ma et al., 2009). Sirotin et al. (2009) provided an alternative explanation for the initial dip, instead of the increase/decrease of the HbO/HbR. According to them, the initial dip can be well explained by the fast increase in the HbT with no increase in the HbR. Similarly, previous studies showed that the neuronal activation generates different spatio-temporal-evoked HR patterns. Each of these patterns becomes dominant at different times and co-localize to a different extent with the neuronal activity (Chen-Bee et al., 2007, 2010). Table 1 presents the studies that specifically focused on the presence and absence of the initial dip using ISOI and some other modalities from years 2003 to 2018.

**Table 1:** Studies that specifically focused on the initial dip in the ISOI and some other modalities (years: 2003–2018, source: Web of Science).

Reference	Modality	Species	Alert/anesthetic	Stimulation	Area
Thompson et al., 2003	O2 microelectrode	Cat	Anesthetic (thiopental)	Visual stimulus	Visual cortex (area 17)
Sheth et al., 2004	ISOI	Rat	Anesthetic (halothane)	Whisker deflection and hindpaw stimulation	Barrel cortex
Thompson et al., 2004	O2 microelectrode	Cat	Anesthetic (thiopental)	Visual stimulus	Visual cortex (area 17)
Suh et al., 2005	ISOI	Rat	Anesthetic (urethane)	Hindlimb stimulation	Neocortex
Sheth et al., 2005	ISOI	Rat	Anesthetic (halothane)	Hindpaw stimulation	Somatosensory cortex
Thompson et al., 2005	O2 microelectrode	Cat	Anesthetic (thiopental)	Visual stimulus	Visual cortex (area 17)
Foster et al., 2005	O2 microelectrode and NADH imaging	Rat	Anesthetic (halothane)	Hypoxia and synaptic activation	Hippocampal slices
Bahar et al., 2006	ISOI	Rat	Anesthetic (urethane)	4-amino-pyridine	Neocortex
Fukuda et al., 2006	ISOI	Cat	Anesthetic (isoflurane)	Visual stimulus	Visual cortex
Suh et al., 2006a	ISOI	Human	Anesthetic (isoflurane and remyfentanyl)	Electrical stimulation	Motor, sensory and language cortex
Chen-Bee et al., 2007	ISOI	Rat	Sodium pentobarbital	Whisker deflection	Somatosensory cortex
Prakash et al., 2007	Optical spectroscopy	Rat and mice	Anesthetic (halothane)	Forepaw stimulation	Somatosensory cortex
Schiessl et al., 2008	ISOI	Monkey	Anesthetic (isoflurane)	Visual stimulus	Visual cortex
Lesage et al., 2009	ISOI	Rat	Anesthetic (isoflurane)	Spinal cord injury	Spinal cord
Ma et al., 2009	ISOI	Human	Anesthetic (isoflurane and remyfentanyl)	Electrical stimulation	Motor, sensory and language cortex
Sirotin et al., 2009	ISOI	Monkey	Alert	Visual stimulus	Visual cortex
Chen-Bee et al., 2010	ISOI	Rat	Sodium pentobarbital	Whisker deflection	Somatosensory cortex
Lee et al., 2012	ISOI and NIRS	Rat	Anesthetic (isoflurane)	Electrical stimulation	Somatosensory cortex
Ma et al., 2016	ISOI	Cat	Anesthetic (isoflurane)	Transcorneal electrical stimulation	Visual cortex
Lu et al., 2017	ISOI	Monkey	Anesthetic (isoflurane)	Moving light spot stimulus	Visual cortex
Sintsov et al., 2017	ISOI	Rat	Anesthetic (isoflurane)	Whisker stimulation	Somatosensory cortex


### Initial Dip in fMRI

The first fMRI study on the initial decrease in the BOLD response was presented in 1995, in which the brain maps of human subjects were found to display a negative change in the signal intensities after the onset of photonic stimulation (Menon et al., 1995). The pixel exhibiting the negative response showed the time course with an initial dip response peaking at approximately 2 s. The authors provided the following interpretation of the BOLD signal that was in good agreement with the initial ISOI studies: at the start of a neuronal activity, the neurons drew oxygen out of the capillary network resulting in the local increase in the paramagnetic HbR that caused the magnetic resonance (MR) intensity to decrease. To compensate for this initial oxygen demand, more oxygenated blood came to the active spot because of the large CBF increase. This increase in the CBF caused the local decrease in the HbR that resulted in an increase in the MR signal.

In comparison to the fMRI study on initial dip, an early study of functional magnetic resonance spectroscopy (fMRS) reported a smaller duration of initial dip and proposed different mechanisms. Menon et al. (1995) suggested that these differences may be caused by the difference in stimulus durations or the partial volume effect. Ernst and Hennig (1994) used a short stimulus duration (0.5 or 1 s), while Menon et al. (1995) used a 10 s stimulus duration. Another study was also conducted by the same fMRS group to further check the effect of different stimulus durations and echo time (TE) on the initial dip (Hennig et al., 1995). They found that the magnitude of the initial dip decreased with the increase of stimulus duration as well as TE, which contradicted the nature of the BOLD signal and did not agree well with the ISOI studies (Hu and Yacoub, 2012). These inconsistencies between the fMRI and fMRS data highlighted the initial dip controversy more.

To follow up on the discrepancies in the fMRI and fMRS data, Hu et al. (1997) performed an fMRI study to further check the effect of stimulus duration (1.5–6 s) on the initial dip. The previous fMRI study by Menon et al. (1995) used a 10 s stimulus duration, but did not examine the dependency of the initial dip on the stimulus duration. The initial dip was detected for all stimulus durations and independent from the stimulus duration for stimuli longer than 3 s. The initial dip magnitude decreased for the short stimulus duration. In comparison to the initial dip magnitude, the magnitude and the rise time of the positive response and the undershoot increased with the increase in the stimulus duration. The initial dip peaked at approximately 2 s and lasted for approximately 4 s depending on the subject. Finally, the ratio of the initial dip peak to the HR peak was approximately 1/3. However, the ratio of the initial dip peak to the HR peak later varied with the strength of the magnetic field of the fMRI system (Janz et al., 1997, 2000; Yacoub and Hu, 1999; Yacoub et al., 2001). All these findings replicate the results of the ISOI studies (Grinvald et al., 1991; Malonek and Grinvald, 1996) and further confirmed the existence of initial dip.

The controversy around the initial dip continued to grow because many studies on animals were unable to detect the initial dip in different brain areas. Some fMRI studies detected the initial dip in the visual cortex of a cat (Duong et al., 2000; Kim et al., 2000a), whereas others were not able to detect any (Jezzard et al., 1997). Similarly, several fMRI studies were not able to detect the initial dip in the somatosensory cortex of rats (Mandeville et al., 1999; Marota et al., 1999; Silva et al., 1999, 2000; Yu et al., 2014), while a study detected the initial dip in the outer layer I in the somatosensory cortex of a rat (Tian et al., 2010). Logothetis et al. (1999) was also able to detect the initial dip in a monkey. Most of the early fMRI studies used the visual cortex to study the initial dip in humans (Hu et al., 1997; Fransson et al., 1998; Yacoub et al., 1999; Janz et al., 2000). Therefore, researchers started to think that the initial dip can only be detected in the visual cortex. The initial dip was later found in the motor cortex (Yacoub and Hu, 2001; Roc et al., 2006; Lindquist et al., 2008) and recently detected in the prefrontal cortex (Kohno et al., 2015). Several studies finally tried to model the relationship between the CBF and the oxygen metabolism to address the controversial initial dips (Buxton et al., 1998, 2004; Robinson et al., 2006; Blanchard et al., 2011; Kim and Ogawa, 2012; Kim et al., 2013; Hadjistassou et al., 2016; Mathias et al., 2017a,b; Angleys et al., 2018); however, the controversy on the presence or interpretation of the initial dip still exists (Uludag, 2008, 2010; Yu et al., 2014).

Table 2 shows the fMRI studies related to the initial dip from years 2003 to 2018. Several studies also continued to report on the initial dip (Yesilyurt et al., 2008; Tian et al., 2010; Tse et al., 2010; Fang and Lee, 2013; Watanabe et al., 2013). Siero et al. (2015) did a very comprehensive study to determine the cortical depth dependence of different HR phases (i.e., initial dip, main HR, and undershoot) in the human visual cortex using 7T-fMRI. They found that the initial dip was dependent on the cortical depth, and the magnitude of the initial dip in the outer cortical region was the largest among the layers. Therefore, they conjectured that the initial dip magnitudes in the deep cortical layers would be very small and may not be detectable without a sufficient signal-to-noise ratio. Schellekens et al. (2017) also recently found the initial dip in the human visual cortex for a single moving bar’s trajectory task. They observed that the amplitude of the positive response and the initial dip in the BOLD signal changed along the motion of the trajectory. No initial dip was present in the BOLD signal when the bar stimulus was near the onset of its motion trajectory. However, the initial dip appeared, and its amplitude increased as the bar stimulus moved closer to the end of its trajectory. They interpreted that the initial oxygen consumption in response to the signaling of a motion stimulus would increase as the stimulus keeps moving. Therefore, they suggested that, at least under some conditions, the initial dip is associated with a neuronal mechanism (i.e., perhaps inhibition).

**Table 2:** fMRI initial dip studies (years: 2003–2018, source: Web of Science).

Reference	fMRI type (field strength)	Species	Alert/anesthetic	Stimulation	Area
Behzadi and Liu, 2006	ASL-fMRI (3T)	Human	Alert	Visual stimulus	Visual cortex
Roc et al., 2006	EPI-fMRI (1.5T)	Human	Alert	Visually guided bilateral hand squeeze task	Motor and visual cortex
Lindquist et al., 2008	Rapid-3D-fMRI (3T)	Human	Alert	Visual-motor and auditory-motor-visual stimulus	Visual, motor, and auditory cortex
Uludag, 2008	EPI-fMRI (3T)	Human	Alert	Visual stimulus	Visual cortex
Yesilyurt et al., 2008	EPI-fMRI (3T)	Human	Alert	Visual stimulus	Visual cortex
Lindquist, 2010	Rapid-3D-fMRI (3T)	Human	Alert	Visual-motor and auditory-motor-visual stimulus	Visual, motor, and auditory cortex
Tian et al., 2010	EPI-fMRI (7T)	Rat	Anesthetic (isoflurane)	Forepaw stimulation	Somatosensory cortex
Tse et al., 2010	EPI-fMRI (3T)	Human	Alert	Visual stimulus	Visual cortex
Fang and Lee, 2013	Optogenetic-fMRI (7T)	Rat	Anesthetic (isoflurane)	Optogenetic stimulation	Motor, hippocampus, and thalamus
Watanabe et al., 2013	EPI-fMRI (3T)	Human	Alert	Visual stimulus and finger tapping task	Visual and motor cortex
Yu et al., 2014	EPI-fMRI (11.7T) Line scanning-fMRI (11.7T)	Rat	Anesthetic (isoflurane)	Forepaw and whisker-pad stimulation	Barrel cortex
Kohno et al., 2015	EPI-fMRI (3T)	Human	Alert	Visual picture stimuli	Ventrolateral prefrontal cortex, visual cortex, and amygdala
Rudrapatna et al., 2015	DT2-fMRI (4.7T) GE3d-EPI-fMRI b-SSFP-fMRI	Rat	Anesthetic (isoflurane)	Spreading depolarization	Whole brain
Siero et al., 2015	EPI-fMRI (7T)	Human	Alert	Visual stimulus	Visual cortex
Lundengard et al., 2016	EPI-fMRI (3T)	Human	Alert	Intensity and frequency visual stimulation	Visual cortex
Schellekens et al., 2017	EPI-fMRI (7T)	Human	Alert	Single moving bar’s trajectory stimulus	Visual cortex


### Initial Dip in fNIRS

Kato et al. (1999) performed the first study that investigated the presence of an initial dip using non-invasive optical imaging (i.e., fNIRS) in humans. They used a 24-channel fNIRS system on the human visual cortex to measure ΔHbO and ΔHbR during a photonic stimulation. They observed an initial increase/decrease in the ΔHbO/ΔHbR after the onset of the stimulation. They claimed that an initial decrease in the ΔHbO is the evidence for the initial oxygen consumption and the contraction of vascular bed during the initial cerebral metabolism. Jasdzewski et al. (2003) later did an in-depth study using differential pathlength factor (DPF) analysis to further investigate the presence of initial dips and HR differences (i.e., onset times and time-to-peak response of HbO, HbR, and HbT) in the fNIRS signals measured from the motor and visual cortices using a rapid-presentation event-related paradigm. They found that the initial dip was present in both visual and motor cortices. The initial dip was clearly observed and did not disappear in the visual data analyzed with extreme DPF values. However, in the case of the motor cortex data, they only found the initial dip for the implausible values of the DPF. They further observed that the HR was delayed by 2 s from the onset. Moreover, the onset of the HbO increase occurred before the HbR decrease for both visual and motor cortices. Therefore, they suggested that different regions in the brain behave differently in relation to the occurrence of initial dips because of different capillary transit times. To further check that the NIRS has the ability to measure the initial dip response, Kato (2004) showed that NIRS has high sensitivity to oxygen exchanges in the capillaries when compared to the fMRI-BOLD signal. Based on this, he demonstrated that NIRS can measure a neuronal-related fast-oxygen response in the capillaries, which is called the fast-oxygen response in capillary event (FORCE), later termed as initial dip. He then suggested that NIRS imaging has a great potential in elucidating the relationship between the initial dip response and the neuronal activity. Akiyama et al. (2006) found a significant increase in the ΔHbR and a decrease in ΔHbO (non-significant) within 1–3 s after task initiation at the center of the primary motor cortex. They also observed that the channel surrounding the center area of the primary motor cortex only showed increase/decrease of the ΔHbO/ΔHbR. Similarly, another study of Wylie et al. (2009) examined the spatiotemporal co-variations among ΔHbO, ΔHbR, and ΔHbT in the visual cortex for their contrast-reversing checkerboard experimental paradigm. They observed a decrease or increase in the ΔHbO/ΔHbR at the start of the activity, which demonstrated the consumption of oxygen at the time of neuronal activity (initial dip) prior to the main HR. Table 3 shows the fNIRS studies that specifically focused on the initial dip response. The studies on the initial dip have slightly increased in recent years. The possible reasons for the increase in the fNIRS studies on the initial dip may be its additional features of simultaneous measurement of the ΔHbO and the ΔHbR, non-invasiveness, and portability. Therefore, it can be said that fNIRS has huge potential as a sensor utilizing the initial dip responses for measuring the neuronal activities in humans and in animals (Lee et al., 2012; Mahmoudzadeh et al., 2017).

**Table 3:** Studies on the initial dip in fNIRS (years: 2003–2018, source: Web of Science).

Reference	Species	Alert/anesthetic	Stimulation	Area	Detection method
Jasdzewski et al., 2003	Human	Alert	Visual stimulus and finger tapping task	Visual and motor cortex	Time-series visualization
Kato, 2004	Human	Alert	Auditory stimulus	Auditory cortex	Time-series visualization
Akiyama et al., 2006	Human	Alert	Hand grasping task	Motor cortex	Time-series visualization
Akin et al., 2006	Healthy and migraine human patients	Alert	Breadth holding task	Prefrontal cortex	Time-series visualization
Wylie et al., 2009	Human	Alert	Visual stimulus	Visual cortex	Time-series visualization
Lee et al., 2012	Rat	Anesthetic (isoflurane)	Electrical stimulation	Somatosensory cortex	Time-series visualization
Yoshino and Kato, 2012	Human	Alert	Single word listening task	Auditory cortex	Vector-based phase analysis
Sano et al., 2013	Human	Alert	Nasal and mouth breathing task	Prefrontal cortex	Vector-based phase analysis
Dutta et al., 2015	Human stroke survivors	Alert	Anodal tDCS stimulation	Central site Cz	Time-series visualization
Hong and Naseer, 2016	Human	Alert	Mental arithmetic and finger tapping tasks	Prefrontal and motor cortex	Vector phase analysis with a threshold circle
Zafar and Hong, 2017	Human	Alert	Mental arithmetic, mental counting, puzzle solving, finger tapping, finger poking, and visual stimulus tasks	Prefrontal, motor, somatosensory, and visual cortex	Vector phase analysis with a threshold circle
Li et al., 2017	Human	Alert	Left and right hand grasping tasks	Motor cortex	SVM classifier
Khan and Hong, 2017	Human	Alert	Mental arithmetic, mental counting, mental rotation and word generation tasks	Prefrontal cortex	LDA classifier
Mahmoudzadeh et al., 2017	Rat	Anesthetic (urethane)	Forepaw stimulation and French male/female words listening task	Somatosensory and auditory cortex	Time-series visualization
Zafar and Hong, 2018	Human	Alert	Right-hand thumb and little finger tapping	Left motor cortex	Vector phase analysis with dual threshold circles


## Detection of the Initial Dip in fNIRS

### Vector Phase Analysis

As observed in Table 3, most fNIRS studies used time series visualization and statistical analysis to discuss the initial dip response. However, Yoshino and Kato (2012) used a method, called vector phase analysis, for the systematic detection of initial dips. The vector phase analysis is a polar coordinate plane method defined by ΔHbO and ΔHbR as orthogonal vector components. Two other vector components [i.e., cerebral oxygen exchange (ΔCOE) and ΔCBV) are obtained by rotating the vector coordinate system defined by ΔHbO and ΔHbR by 45° counterclockwise using the following equations (Yoshino et al., 2013).

$$\Delta CBV=\frac{1}{\sqrt{2}}\left(\Delta HbO+\Delta HbR\right)$$

$$\Delta COE=\frac{1}{\sqrt{2}}\left(\Delta HbR-\Delta HbO\right)$$

The value of ΔCBV is slightly lower than ΔHbT, since ΔHbT is calculated as the sum of ΔHbO and ΔHbR as follows.

ΔHbT=ΔHbO+ΔHbR

Using Eqs. (1) and (3), the relationship between ΔCBV and ΔHbT is represented as (Oka et al., 2015).

$$\Delta CBV=\sqrt{2}\Delta HbT$$

The magnitude and the phase of a vector *p* = (ΔHbO, ΔHbR) in the phase plane can be calculated as follows.

$$|p|=\sqrt{\Delta {HbO}^{2}+\Delta {HbR}^{2}}$$

$$∠p=\tan^{-1}(\frac{\Delta HbR}{\Delta HbO})=\tan^{-1}(\frac{\Delta COE}{\Delta CBV})+45^{{}^{\circ}}$$

**FIGURE 3:**
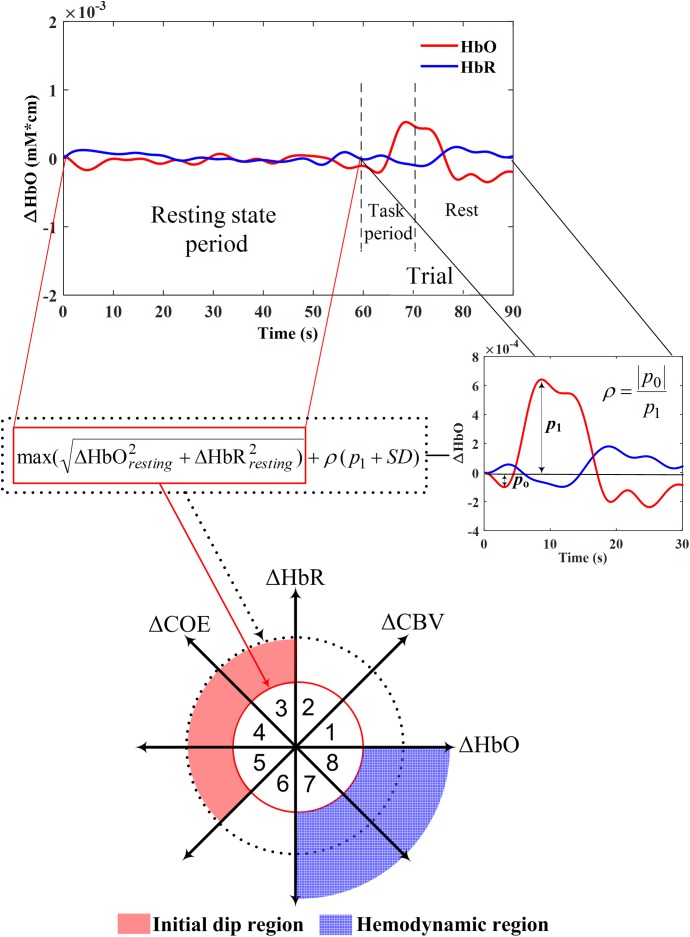
Vector phase diagram with threshold circles (Yoshino and Kato, 2012; Hong and Naseer, 2016; Zafar and Hong, 2018).

The ratio of ΔCOE to ΔCBV (i.e., ΔCOE/ΔCBV) defines the degree of oxygen exchange. Therefore, ΔCOE represents the oxygen exchange in the blood vessels and thus also the neuronal activities (Oka et al., 2015). ΔCOE > 0 indicates that deoxygenation is occurring in the capillaries as a result of oxygen consumption by the nerve cells and, therefore, represents hypoxia in the blood vessels. On the other hand, ΔCOE < 0 indicates that the oxygen-containing red blood cells are being supplied by the arteries and, thus, a high level of oxygenation in the blood vessels. Figure 3 shows the phase diagram and its decomposition into eight phases/regions. Table 4 summarizes the description and interpretations of these eight phases/regions. Phases 1 to 5 in Table 4 are the initial dip phases because they reflect an increase in either ΔHbR or ΔCOE. Therefore, an event-related vector residing in these regions is defined as an initial dip. The increases of ΔCBV and ΔHbR in Phases 1 and 2 (i.e., mostly observed in the fMRI and ISOI) are called the hyperemia dip phases (Malonek and Grinvald, 1996; Jones et al., 2001; Hu and Yacoub, 2012). Phases 3–5 are the hypoxic dip phases with a decrease in the ΔHbO. Phase 3 is called the hypoxic-hyperemia dip caused by the increase in the ΔCBV, whereas Phases 4 and 5 are categorized as the hypoxic-ischemia dip caused by the decrease in the ΔCBV. It is noted that Phases 1–3 occur in all three modalities (i.e., ISOI, fMRI, and fNIRS). ISOI and fMRI initial dip studies were mostly focused on the initial increase in the ΔHbR and ΔCBV (Sirotin et al., 2009). In these three phases (i.e., 1–3) both the ΔHbR and ΔCBV increase enabling the vector phase analysis to detect the ISOI and fMRI initial dips. However, in Phases 4 and 5, the ΔCBV is decreasing, which cannot be visualized/discussed in ISOI and fMRI (Sintsov et al., 2017). As fNIRS simultaneously measured ΔHbO and ΔHbR, the decrease in ΔCBV can possibly be detected, which enables fNIRS to detect five types of initial dips. Out of these five initial dip phases, the hypoxic dip phases (i.e., Phases 3–5, in which ΔCOE > 0) frequently occurred in the fNIRS signals (Sano et al., 2013; Zafar and Hong, 2017). Phases 3–5 indicate a hypoxic change in the blood vessels, thereby representing deoxygenation in the capillaries. Both ΔHbR and ΔCOE decrease in Phases 6–8, and these are called non-dip phases. In comparison to the non-dip phases, neuroactivation in the initial dip phases is considered higher (Yoshino and Kato, 2012).

**Table 4:** Phase division of the vector diagram.

Phases	Conditions	Description	Modalities in which a specific phase can possibly occur
1	0 < ΔHbR < ΔHbO, ΔCOE < 0 < ΔCBV	Hyperemia dip phase with ΔHbR > 0	ISOI, fMRI, and fNIRS
2	0 < ΔHbO < ΔHbR, 0 < ΔCOE < ΔCBV		ISOI, fMRI, and fNIRS
3	ΔHbO < 0 < ΔHbR, 0 < ΔCBV < ΔCOE	Hypoxia-hyperemia dip phase with ΔCOE > 0	ISOI, fMRI, and fNIRS
4	ΔHbO < 0 < ΔHbR, ΔCBV < 0 < ΔCOE	Hypoxia-ischemia dip phase with ΔCOE > 0	fNIRS
5	ΔHbO < ΔHbR < 0, ΔCBV < 0 < ΔCOE		fNIRS
6	ΔHbR < ΔHbO < 0, ΔCBV < ΔCOE < 0	Non-dip phases with ΔCOE < 0	fNIRS
7	ΔHbR < 0 < ΔHbO, ΔCOE < ΔCBV < 0		fNIRS
8	ΔHbR < 0 < ΔHbO, ΔCOE < 0 < ΔCBV		ISOI, fMRI, and fNIRS


**ISOI, intrinsic signal optical imaging; fMRI, functional magnetic resonance imaging; fNIRS, functional near-infrared spectroscopy*.*

**FIGURE 4:**
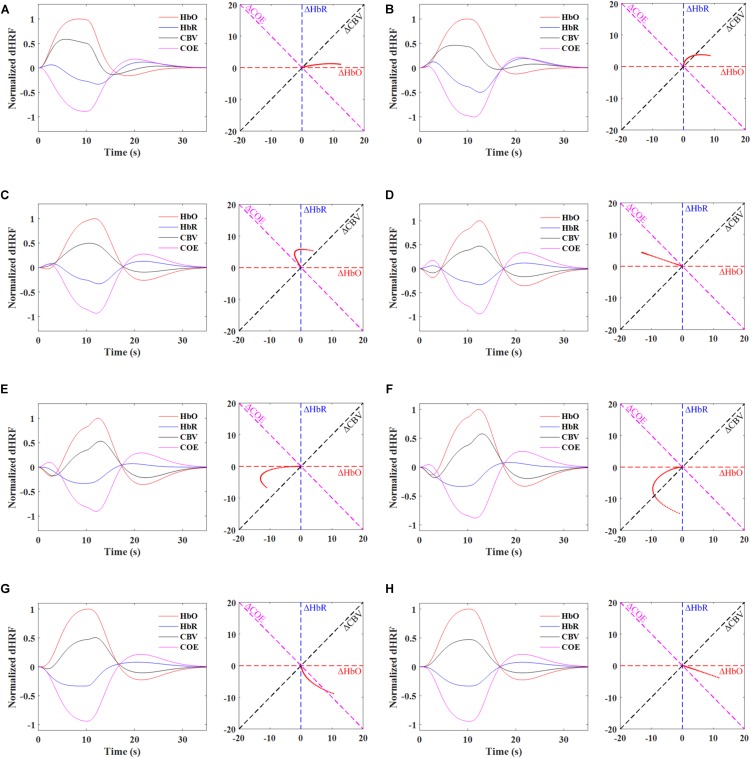
Phase diagrams depicting the ideal trajectories (0 to 4 s) of individual phases using dHRF-based HbO and HbR, Phases 1∼5 represents the initial dip response and Phases 6 and 7 denote the conventional hemodynamics response: **(A)** Phase 1, 0 < ΔHbR < ΔHbO, ΔCOE < 0 < ΔCBV, **(B)** Phase 2, 0 < ΔHbO < ΔHbR, 0 < ΔCOE < ΔCBV, **(C)** Phase 3, ΔHbO < 0 < ΔHbR, 0 < ΔCBV < ΔCOE, **(D)** Phase 4, ΔHbO < 0 < ΔHbR, ΔCBV < 0 < ΔCOE, **(E)** Phase 5, ΔHbO < ΔHbR < 0, ΔCBV < 0 < ΔCOE, **(F)** Phase 6, ΔHbR < ΔHbO < 0, ΔCBV < ΔCOE < 0, **(G)** Phase 7, ΔHbR < 0 < ΔHbO, ΔCOE < ΔCBV < 0, and **(H)** Phase 8, ΔHbR < 0 < ΔHbO, ΔCOE < 0 < ΔCBV.

Synthetic ΔHbO and ΔHbR trials were generated with the designed hemodynamic response function (dHRF) assuming a trial period of 35 s (i.e., 10 s task and 25 s rest) to further elaborate the vector phase analysis. The dHRF-HbO was modeled by convolving three gamma functions with the trial period. Similarly, dHRF-HbR was generated by multiplying dHRF-HbO with a -1/3 factor (Pinti et al., 2017). Figure 4 shows the time-domain signals of ΔHbO, ΔHbR, ΔCOE, ΔCBV and the trajectories of individual phases for the time period from 0 to 4 s. The time domain signals help to visualize the trajectory of each phase. For example, in Phases 1 and 2, there is no initial decrease in the ΔHbO but, at the same time, ΔHbR increases representing the initial consumption of oxygen. Similarly, there is no initial increase in ΔHbR in Phase 5 but an initial decrease in ΔHbO showing the initial oxygen consumption. Phases 3 and 4 are the most likely and appropriate representation of the initial dip because the ΔHbO and ΔHbR are decreasing and increasing at the same time. It is also important to note that the trajectory of a correct single trial HR (having initial dip) will initially remain in Phases 1–5 within first 2–4 s, and then it will move to the non-initial dip phases (i.e., 6–7 s), after 2–4 s. Table 4 and Figure 4 present that the vector phase analysis includes all possible interpretations of the initial dips that can possibly be observed in the ISOI, fMRI, and fNIRS modalities. Another advantage of the vector phase analysis is that the ΔCOE vector cannot easily be affected by the changes in the ΔCBV or skin blood flow because the ΔCBV is perpendicular to ΔCOE [please see the details in Oka et al. (2015)]. Therefore, the ΔCOE can be a better physiological indicator of increased brain functionality compared to the conventionally used ΔHbO and ΔHbR.

### Threshold Circle Criterion in the Vector Phase Analysis

#### Single Threshold Circle Criterion

The main issue in the vector phase analysis of Yoshino and Kato (2012) was that an unrelated large fluctuation of ΔHbO and ΔHbR in the resting state and even during the task period might be interpreted as an initial dip. Moreover, the detection time of an initial dip was not specified in the vector diagram. This issue was further addressed by Hong and Naseer (2016) by introducing a threshold circle (red circle, Figure 3) in the vector diagram. The radius of the threshold circle was determined during the resting state period as follows.

$$r_{1}=\max\left(\sqrt{\Delta {HbO}_{resting}^{2}+\Delta {HbR}_{resting}^{2}}\right)$$

They proposed that this threshold circle can be used as a decision criterion for the initial dip occurrence. Their criteria for initial dip detection were (i) the trajectory must lie in any of Phases 2–5 (i.e., ΔCOE > 0), and (ii) the magnitude of the trajectory should deviate from the threshold circle. They also found the time of initial dip occurrences using the threshold circle in the vector diagram. Because of the inherent onset delay in fNIRS, they also proposed the usage of an auto-regressive moving average model with an exogenous input in combination with the vector phase analysis method to predict, *q*-steps ahead, the occurrence of initial dips. They were able to reduce the time lag in detecting an initial dip to approximately 0.9 s. The single threshold circle criteria in the vector phase diagram worked significantly well to reducing the detection time of brain activity in fNIRS BCI. However, large fluctuations of ΔHbO and ΔHbR above the threshold circle during the task period can still be interpreted as an initial dip.

#### Dual Threshold Circles Criterion

Zafar and Hong (2018) recently proposed to use a secondary circle as an upper bound for initial dip detection (black dotted circle in Figure 3) in the vector phase analysis to further reduce the false detection of initial dip. The radius of the second threshold circle was defined as follows.

$$r_{2}=r_{1}+\rho \left(p_{1}+SD\right)$$

where *p*_1_ and SD, respectively, are the amplitude and the standard deviation of the conventional HR, and ρ is the ratio of the amplitude of the initial dip (*p*_0_) and *p*_1_. The value of ρ, *p*_1_, and SD can be determined through the averaging of the HRs over several trials from the most active channel for a given task. Once the experimental data is obtained, the radius (*r*_2_) can be calculated based on the averaged data and its SD for the specific tasks, measured locations, and subjects.

In Zafar and Hong (2018), the value of ρ was set to 0.3 using the empirical data. Moreover, in the case of no initial dip detection due to averaging or noises, the ratio was still kept to 0.3 for the second threshold circle. They defined more specific regions for the hypoxic initial dip and the conventional HR on the vector phase diagram using the dual threshold circles. The hypoxic initial dip region (i.e., the red shaded area in Figure 3) was defined as the region between the two threshold circles in Phases 3–5. Similarly, the outer region of the first threshold circle in Phsaes 7 and 8 was defined as the HR region (i.e., the blue dotted area in Figure 3). The requirement that the trajectory must lie within the two threshold circles in Phases 3–5 was the proposed criterion for the detection of initial dip. Any trajectory going outside the secondary threshold circle was considered as a false dip or noise. Finally, if the trajectory remains within the two threshold circles in Phases 3–5 within first 2–4 s period and it moves to either Phase 7 or 8 after 2–4 s, it was considered as a correct trajectory of HR upon the given trial including the initial dip. Using the dual threshold circles in the vector phase analysis, they were able to separate the false dip channels among the candidate channels found with the single threshold circle criteria, thereby improved the detection percentage of initial dips.

### dHRF-Based Detection of Initial Dip

The detection of initial dip in the HR can also be done using an initial-dip-based dHRF, which can be generated by convolving the canonical hemodynamic response function (cHRF) made with three gamma functions, denoted by h(k), with the stimulus period, u(k), as follows.

$$dHRF(k)=\overset{k-1}{\underset{n=0}{\Sigma }}h(n)u\left(k-n\right)$$

$$u(k)=\left\{ \begin{array}{c} 1,if\,\;k\in task,\\ 0,if\,\;k\in rest, \end{array}\right.$$

where task and rest represent the task period and the rest period, respectively. Recalling that the most frequently used option for the modeling of cHRF is a gamma function (Friston et al., 1994, 1998), the following three gamma functions can be used to model the cHRF including the initial dip, HR, and undershoot (Shan et al., 2014).

$$h(k)=\overset{3}{\underset{i=1}{\Sigma }}A_{i}\frac{k^{a_{i}-1}\beta_{i}^{a_{i}}e^{-\beta_{i}k}}{\Gamma \left(\alpha_{i}\right)}$$

where *i* represents the number of gamma functions, *A_i_* is the amplitude, α*_i_* and β*_i_* tune the shape and the scale, respectively, and *k* is the time step. Then, *t*-statistics analysis can be used to estimate the initial dip in the HR by fitting the measured HR to the initial-dip-based dHRF. Please see Friston et al. (2007), Lindquist et al. (2009), Hong et al. (2018b) for details.

## Factors Affecting the Initial Dip

Some possible contributing factors in the elusive nature of initial dip could be from different species, anesthesia, different methodologies adopted, surgical procedures, and stimulation protocols [please see Ances (2004), Vanzetta and Grinvald (2008), and Hu and Yacoub (2012) for further details]. The difference in species can affect the dynamics in the HRs. Prakash et al. (2007) showed that the HRs caused by the functional stimulation were different in the neocortices of mice and rats. The authors concluded that the HRs under a physiological stimulation can differ in the species because of different cortical architectures. In addition, several studies in ISOI and fMRI on the rat somatosensory cortex were not or scarcely able to detect the initial dip. A possible reason for this might be that smaller animals like rats have a higher blood flow and a subsequently lower cerebral transit time because of their shorter mean capillary length, which results in a small or no initial dip (Ances, 2004; Vanzetta and Grinvald, 2008). Similarly, several studies showed that the degree of anesthesia and anesthetic versus alert conditions can affect the initial dip magnitude in rats (Berwick et al., 2002) and monkeys (Shtoyerman et al., 2000). The initial dip magnitudes in awake animals were significantly larger than those in anesthetized animals. The degree of anesthesia can affect metabolism and blood flow, which in turn affects the magnitude of initial dip (Jones et al., 2001). In addition, the amount of oxygen in the blood (i.e., oxygen blood saturation) and the hypercapnia level may influence the initial dip amplitude (Hyder et al., 1998, 2000; Mayhew et al., 2001). Similarly, Fukuda et al. (2006) enhanced the initial dip in a cat visual cortex by reducing the arterial blood flow pressure. Furthermore, caffeine, which is a vasoconstrictor that can affect the blood flow response, reduces the initial dip magnitude in humans (Behzadi and Liu, 2006). Finally, different stimulation protocols may induce different metabolic demands that can influence the amount of HbO/HbR in the microcirculation, thereby resulting in different HRs (Vanzetta and Grinvald, 2001). Schellekens et al. (2017) recently showed that the neuronal activity can be directly associated with specific stimulus features, which can be the main possible reason for the elusiveness of the initial dip. In summary, several physiological factors can affect the existence of the initial dip and contribute to the discrepancies and elusive nature of initial dip.

## Role of the Initial Dip in BCI

In general, a BCI scheme includes (i) signal acquisition and preprocessing, (ii) feature extraction, (iii) classification, and (iv) feedback. In the HR-based imaging for BCI, the detection of HRs (i.e., the increase of ΔHbO) is the main focus on neuronal command interpretation (Matthews et al., 2008). To the best of authors’ knowledge, ISOI has not been used for BCI applications so far. However, fMRI and fNIRS were used for many non-invasive BCI applications.

### fMRI-Based BCI

With the technical advances in MRI, a real-time fMRI can work as a closed-loop system that allows simultaneous acquisition, analysis, and visualization of brain images in real-time (Cox et al., 1995; Sitaram et al., 2007). The univariate and multivariate methods were used for the analysis of fMRI images in real-time. In univariate analysis, the brain activity is measured from thousands of brain locations repeatedly and then each location is analyzed separately for decoding perceptional or cognitive tasks (Haynes and Rees, 2006). The main objective of the univariate analysis is to determine the voxels that are significantly correlated with a specific task. The univariate methods include the real-time correlation and general linear model analysis (Gembris et al., 2000; Bagarinao et al., 2003). In contrast to univariate methods, multivariate or pattern-based methods take into account the pattern information of the brain activity measured simultaneously at many locations (Cox and Savoy, 2003; Haynes and Rees, 2005b; Kriegeskorte et al., 2006). Pattern-based methods use voxel intensities and their spatiotemporal relationships as features to decode the brain activity (Kamitani and Tong, 2005; Sitaram et al., 2008). The frequently used classifiers in pattern-based methods include multilayer neural networks (Norman et al., 2006), linear discriminant analysis (LDA) (Carlson et al., 2003; Haynes and Rees, 2005a; O’toole et al., 2005), support vector machine (SVM) (LaConte et al., 2005, 2007; Mourao-Miranda et al., 2005), Gaussian naive Bayes classifiers (Mitchell et al., 2004), and correlation-based classifiers (Haxby et al., 2001; Spiridon and Kanwisher, 2002). Finally, the output of a classifier is feedbacked to help the participants/patients to exercise voluntary self-regulation on the specific brain area. The feedback can be presented in the form of visual (deCharms et al., 2004; Chiew et al., 2012; Sitaram et al., 2012; Veit et al., 2012; Zapaa et al., 2018), auditory (Posse et al., 2003; Yoo et al., 2006), and virtual reality (Lorenzetti et al., 2018).

Several studies successfully demonstrated that the participants using real-time feedback in fMRI-BCI were able to perform voluntary self-regulation on a focused brain region, see details in Ruiz et al. (2014) and Sorger et al. (2018). The applications of real-time neuro-feedback in fMRI-BCI for healthy subjects include the voluntary self-regulation of motor areas (Yoo and Jolesz, 2002; Yoo et al., 2004, 2008; Berman et al., 2012; Johnson et al., 2012), sensory areas (Scharnowski et al., 2012; Robineau et al., 2014; Auer et al., 2015), auditory cortex (Yoo et al., 2006), language areas (Rota et al., 2009), emotion areas (Weiskopf et al., 2003; Caria et al., 2007, 2010; Zotev et al., 2011), and working memory areas (Zhang et al., 2013; Sherwood et al., 2016). In the case of patients, the real-time neuro-feedback helps to remediate the pathological brain activation associated with different disorders including neurological disorders (Subramanian et al., 2011; Sitaram et al., 2012; Guan et al., 2015) and psychiatric disorders (Ruiz et al., 2013; Hartwell et al., 2016; Kirsch et al., 2016; Zotev et al., 2016). However, all the real-time neuro-feedback fMRI-BCI studies used the increasing positive HR/BOLD response to provide feedback to the participants, which limits its temporal resolution due to a time lag between the neuronal activity and the detected BOLD response (Sitaram et al., 2007). So far, no study has used the initial dip response of the HR/BOLD to provide immediate feedback to the participants. The initial dip can help to reduce the neurofeedback time in fMRI-BCI. Another major limitation of fMRI-BCI is the bulky hardware and the restrictive environment that prevents fMRI-BCI to be used as a portable device, thereby making fMRI-BCI unsuitable for the routine use.

### fNIRS-Based BCI

Functional near-infrared spectroscopy has so far shown great potential for use as a portable device for BCI applications (Park et al., 2016; Yin et al., 2016; Chaudhary et al., 2017; Huang et al., 2017; Sereshken et al., 2017). However, the main issue associated using fNIRS signals for BCI applications is the inherent 2 s time delay from the neuronal activation (Jasdzewski et al., 2003; Hong and Nguyen, 2014). Therefore, researchers in the fNIRS community employed various features in 0–5, 2–7, 0–10, 0–15, 0–17, and 0–20 s time windows to classify the HRs from the same or different brain regions using multi-class classification algorithms (Power et al., 2011; Khan et al., 2014; Schudlo and Chau, 2014; Gateau et al., 2015; Khan and Hong, 2015; Hong and Santosa, 2016; Hong et al., 2017; Liu and Hong, 2017; Shin et al., 2017). The commonly used HR (i.e., ΔHbO and ΔHbR) features include the signal mean, signal peak, signal slope, skewness, kurtosis, variance, standard deviation, number and sum of peaks, root mean square, and median (Hwang et al., 2016; Naseer et al., 2016a; Hong et al., 2018b). The signal mean, signal peak, and signal slope in the 2–7 s (i.e., 5 s) windows from the onset were found to yield better classification accuracies for fNIRS-BCI using HRs (Hong et al., 2015; Naseer and Hong, 2015). Like fMRI, the frequently used classifiers for the fNIRS features discrimination include LDA, SVM, extreme machine learning, Bayes classifiers, and neural networks (Chan et al., 2012; Yin et al., 2015; Bui et al., 2016; Kim et al., 2016; Naseer et al., 2016b; Ding et al., 2017).

Zafar and Hong (2017) recently addressed the issue of the inherent delay by applying the initial dip detection method (i.e., the vector phase analysis) through changing the threshold circle of Hong and Naseer (2016) from max(ΔHbO^2^+ ΔHbR^2^)^1/2^ to max{ΔHbO, ΔHbR} to the classification problem of three mental tasks that originated from the prefrontal cortex for BCI. They examined five features of ΔHbO during the initial dip phase: signal mean, signal minimum, signal peak, skewness, and kurtosis in different window sizes (i.e., 0–1, 0–1.5, 0–2, and 0–2.5 s) to classify multiple tasks from the prefrontal cortex in an offline analysis. They concluded that the signal mean and the signal minimum as features for the initial dip worked well in the 0–2.5 s window to classify three prefrontal tasks using the LDA as a classifier. They demonstrated that the moving window size in the fNIRS-based BCI can be reduced to 2.5 s using the initial dip detection method. Similarly, another study used the mean value of ΔHbO and ΔHbR in the 0–2 s window as an initial dip feature for the classification of left- and right-hand movements (Li et al., 2017). They were able to attain a higher classification accuracy of 85.5% using SVM as a classifier for the two-class BCI in a reduced window size of 0–2 s. Khan and Hong (2017) also achieved an LDA-based high classification accuracy of 75.6% in a reduced window size (i.e., 0–2 s) for a four-class fNIRS-BCI using the signal minimum as an initial dip feature. Recently, the improvement in the classification accuracies using dual threshold circles in the vector phase diagram and the use of three gamma functions for online fNIRS-BCI using initial dip has been proposed. Table 5 presents more details on fNIRS-BCI studies using the initial dip.

**Table 5:** Functional near-infrared spectroscopy (fNIRS)-BCI studies using the initial dip.

Reference	Paradigm	Brain area	Features	Window size	Classifier	Commands	Accuracy
Zafar and Hong, 2017	Mental arithmetic, mental counting, and puzzle solving tasks	Prefrontal cortex	Signal mean, minimum, signal slope, skewness, and kurtosis of ΔHbO	2.5 s	LDA	3	57.5% using signal mean and minimum values
Li et al., 2017	Left and right hand grasping tasks	Motor cortex	Mean ΔHbO and ΔHbR for fNIRS and wavelet coefficient for EEG	2 s	SVM	2	85.5% for fNIRS and 91.0% for hybrid EEG-fNIRS
Khan and Hong, 2017	Mental arithmetic, mental counting, mental rotation and word generation tasks	Prefrontal cortex	Signal peak, minimum, and signal mean of ΔHbO	2 s	LDA	4	75.6%
Zafar and Hong, 2018	Right-hand thumb and little finger tapping	Left motor cortex	Signal mean and minimum value of ΔHbO	2.5 s	LDA	2	74.9%


**LDA, linear discriminant analysis; SVM, support vector machine*.*

## Discussion and Future Implications

**FIGURE 5:**
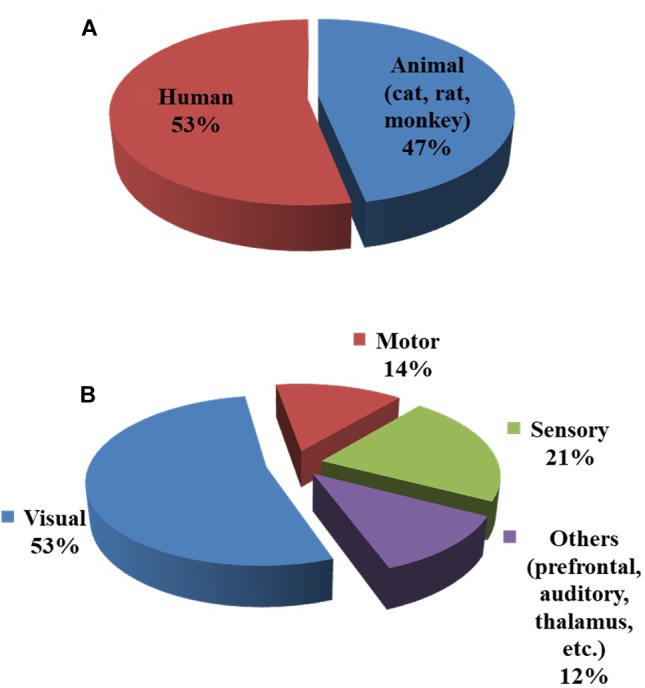
Subjects and brain areas used in initial dip studies: **(A)** human vs. animal (cat, rat, and monkey) subjects, **(B)** brain areas. The charts were constructed using 103 articles (1990–2018) from Web of Science (www.isiknowledge.com).

**FIGURE 6:**
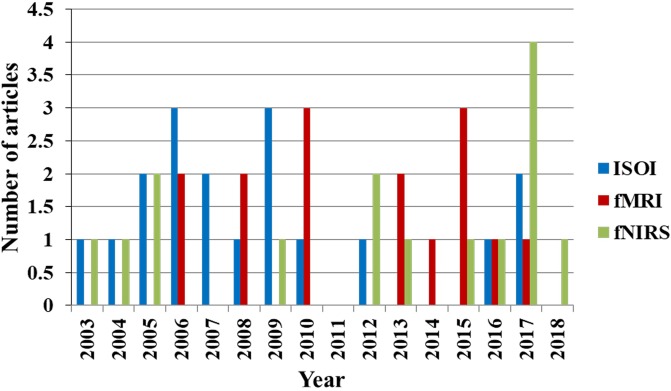
Trend of publications on initial dip in ISOI, fMRI, and fNIRS (source: Web of Science).

As observed in Figure 2, the initial dip has been studied and detected in various modalities. Various subjects (i.e., human and animal species) and brain areas have been considered in these studies. Figure 5 shows the percentage breakdown of these studies. Therefore, the question whether the detected initial dip is/was a noise or artifact is very unlikely. The discrepancy in the findings or the elusive nature of the initial dip may be caused by methodological or physiological differences. Although the initial dip has shown potential in ISOI and fMRI for mapping the orientation columnar structure, the reliability, reproducibility, and interpretation of the initial dip have been argued for several years (Kim et al., 2000b; Logothetis, 2000; Sirotin et al., 2009; Uludag, 2010; Uludag and Blinder, 2018). Studies continued reporting about the initial dip (Tables 1, 2), but the interests in the initial dip studies in ISOI and fMRI have significantly faded over the years because of the inability to make wide use of it (see Figure 6), especially in humans.

The BCI so far seems to be a potential application for the initial dip utilization. The main objective of the BCI is to translate the brain signal into commands instead of understanding the brain signals in terms of NVC or diagnosis of a brain disease. The three research issues in the BCI field are as follows: (i) how to increase the classification accuracy, (ii) how to increase the number of brain commands for improving the degrees of freedom of an external device, and (iii) how to quickly decode the brain commands by reducing the delay. The initial dip can help address two out of these three issues for the BCI. First, the initial dip detection can reduce the detection time (i.e., window size for BCI). Second, the generation of brain commands from a restricted brain region can become diverse, which consequentially results in an increased number of commands from a wider brain region, because the initial dip is spatially specific to regional neuron firing. The previous fNIRS studies (Table 5) showed the application of the initial dip in the BCI. Although these studies demonstrated that the utilization of the initial dip detection can reduce the window size to 2 or 2.5 s for fNIRS-BCI, significant research is still needed to improve the method of analysis and signal-to-noise ratio of fNIRS signals to achieve a better initial dip detection.

Previous fNIRS-BCI studies only used the constant DPF analysis to detect the initial dip. Jasdzewski et al. (2003) showed that the initial dip did not disappear when using the extreme DPF analysis in the visual cortex. It also only appeared in the motor cortex for plausible DPF values. Zafar and Hong (2017) found the initial dip in the prefrontal, motor, and visual cortices with the constant DPF analysis, but did not observe the initial dip in the somatosensory cortex. Similarly, most of the studies on the somatosensory cortex of a rat did not also detect the initial dip, with a possible reason of small capillary transit time. The detection of the initial dip in human somatosensory should be further investigated, and would be helpful in identifying the specific brain area in somatosensory that can help in restoring sensation in amputees (Ghafoor et al., 2017). More research is required to check the effect of DPF values on the initial dip in fNIRS signals obtained from different brain areas. This DPF analysis will help in fNIRS brain imaging to distinguish the capillary vascular responses.

**FIGURE 7:**
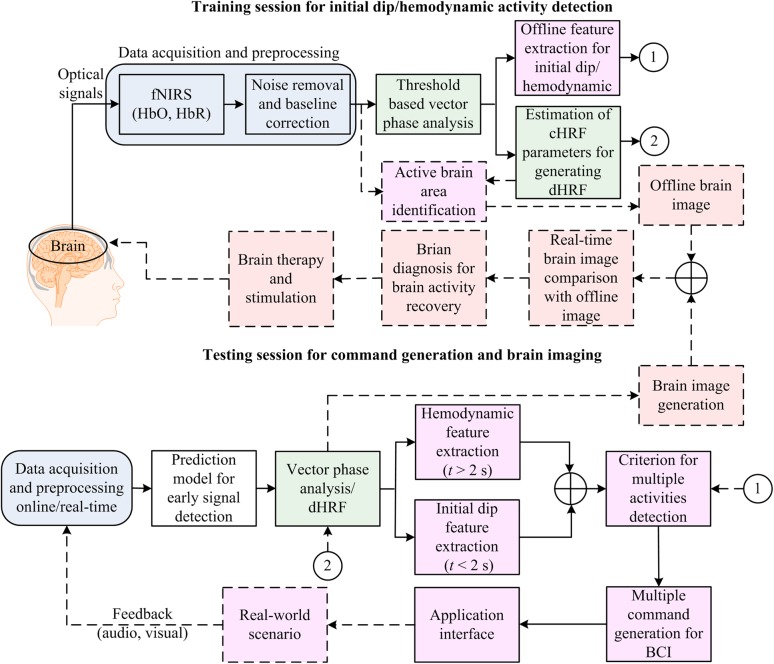
Brain–computer interface (BCI) framework: application of the initial dip detection.

Figure 7 illustrates the proposed BCI framework incorporating the initial dip detection. The main difference from the conventional scheme that uses the HRs is that even though the initial dip detection procedure fails, the conventional scheme still backs up. A fail-tolerant loop must be considered in the feature selection and classification for online control command generation. The major concern to be addressed in the future is the improvement of the initial dip detection for fNIRS-BCI applications. The vector-phase analysis with a dual threshold circle is one of the available options. However, future research is needed to determine the optimal radius for the threshold circles. Similarly, for online fNIRS-BCI, an extensive future work is required to determine the best possible functions (e.g., gamma function, Gaussian function, half cosine function, etc.) for modeling the initial-dip-based dHRF. The possible utilization of the initial dip in brain diagnosis and therapy is also proposed in Figure 7 because some previous studies found initial dip in patients (Akin et al., 2006; Suh et al., 2006a; Dutta et al., 2015).

The delay between the oxygen consumption and the additional blood flow results in the form of initial dip. It is not possible for the additional blood to arrive in from a too distal area to the epicenter of activation within a very short period of time (Hadjistassou et al., 2016), but it comes from nearby. This delay can vary from 0.1 to 2 s (Buxton et al., 2004). Several previous studies have reported that the peak of an initial dip occurs at approximately 2–2.5 s and the dip phase completes around 3.5–4.5 s (Hu and Yacoub, 2012; Zafar and Hong, 2017). Therefore, the selection of features and window size for the classification of initial dip for fNIRS-BCI also needs much attention. Table 5 shows that the signal mean of ΔHbO/ΔHbR and the signal minimum of ΔHbO extracted in the 0–2 or 0–2.5 s window are mostly used as features for the initial dip classification. Li et al. (2017) were able to achieve high classification accuracy in a reduced window (i.e., 2 s) using hybrid EEG-fNIRS. Therefore, the use of EEG-NIRS, fast optical response (Hu et al., 2011), and adaptive signal processing algorithms (Hu et al., 2010, 2013; Hamadache and Lee, 2017; Song et al., 2017; Li et al., 2018) can help to reduce the inherent delay in the HR, which further result in a possible reduction of the window size. Also, in the future, other features, including ΔHbR, ΔCOE, and ΔHbT should be investigated for further improvement of the initial dip classification accuracy. Another interesting issue that should be addressed in the future is the initial dip appearing in ΔHbT, which can be more reliable and spatially specific to the neuronal activity site as compared to ΔHbO or vice versa.

Finally, the current need is to understand how the brain works and how these neuroimaging modalities can be helpful for mankind. Considering that fMRI has high spatial, but low temporal resolution, further research by combining neuronal modalities (e.g., EEG) with the HR modalities might be a more promising brain diagnostic endeavor. The study by Dutta et al. (2015) demonstrated that the initial dip in HbO due to anodal transcranial direct current stimulation was associated with an increase in the log-transformed power of EEG within 0.5–11.25 Hz frequency band in stroke patients. Therefore, in the future, the initial dip phenomenon might be well addressable by combining EEG either with fNIRS or fMRI (Shah et al., 2013; Hong and Khan, 2017; Mano et al., 2017). In summary, all recent advancements or findings using non-invasive modalities like EEG, fMRI, and fNIRS are adding information toward a better understanding of the brain.

## Conclusion

The ultimate goals of these HR-based neuroimaging modalities are (i) to provide an understanding of the NVC and (ii) how these modalities can be used as a means of communication to disabled persons, resulting in the betterment of humanity. Meanwhile, the spatial and temporal characteristics of the HR as an alternative to the neuronal activity are still being debated. Therefore, in this article, we presented a review on the existence and the elusive nature of the initial dip duration of HRs in ISOI, fMRI, and fNIRS. We discussed the brief story of the initial dip and the beginning of the controversy regarding the presence of the initial dip in ISOI/fMRI. We also presented the detection and the role of the initial dip in the brain–computer interface using fNIRS.

The initial dip was successfully detected in ISOI, fMRI, and fNIRS. Other techniques like phosphorous quenching and direct tissue-oxygen O_2_ microelectrode also showed evidence for prior oxygenation before the increase in the CBF that further confirmed the existence of the initial dip. Therefore, the detected initial dip using the abovementioned modalities is not likely to be an artifact. However, the discrepancy in detecting the initial dip is most likely caused by methodological, physiological, and modality differences.

Furthermore, the BCI seems to be a potential application for the initial dip utilization. Despite the low spatial resolution of fNIRS compared to ISOI and fMRI, fNIRS can be used for BCI applications with the advantage of providing simultaneous information on oxyhemoglobin and deoxy-hemoglobin, portability, and low cost. The use of the initial dip can help reduce the window size for generating the brain commands for BCI. Initial dips can also help increase the number of commands from a wide area because they are more spatially specific to the neuronal sites. Therefore, research on the initial dip must be continued, and more sophisticated methods of analysis must be developed to reduce the elusiveness surrounding the initial dip.

## Author Contributions

K-SH conceived the topic and finalized the paper. AZ conducted the literature survey and wrote a preliminary version.

## Conflict of Interest Statement

The authors declare that the research was conducted in the absence of any commercial or financial relationships that could be construed as a potential conflict of interest. The reviewer NN declared a past co-authorship with one of the authors K-SH to the handling Editor.
